# *Burkholderia mallei* and *Burkholderia pseudomallei* stimulate differential inflammatory responses from human alveolar type II cells (ATII) and macrophages

**DOI:** 10.3389/fcimb.2012.00165

**Published:** 2012-12-28

**Authors:** Richard Lu, Vsevolod Popov, Jignesh Patel, Tonyia Eaves-Pyles

**Affiliations:** ^1^Department of Microbiology and Immunology, University of Texas Medical BranchGalveston, TX, USA; ^2^Department of Pathology, University of Texas Medical BranchGalveston, TX, USA

**Keywords:** *Burkholderia*, alveoli, epithelial cells, macrophages, cytokines, innate immune response, Toll-like receptor

## Abstract

Alveolar type II pneumocytes (ATII) and alveolar macrophages (AM) play a crucial role in the lung's innate immune response. *Burkholderia pseudomallei* (*BP*) and *Burkholderia mallei* (*BM*) are facultative Gram-negative bacilli that cause melioidosis and glanders, respectively. The inhalation of these pathogens can cause lethal disease and death in humans. We sought to compare the pathogenesis of and host responses to *BP* and *BM* through contact with human primary ATII cells and monocytes-derived macrophages (MDM). We hypothesized that because *BP* and *BM* induce different disease outcomes, each pathogen would induce distinct, unique host immune responses from resident pulmonary cells. Our findings showed that *BP* adhered readily to ATII cells compared to *BM*. *BP*, but not *BM*, was rapidly internalized by macrophages where it replicated to high numbers. Further, *BP*-induced significantly higher levels of pro-inflammatory cytokine secretion from ATII cells (IL-6, IL-8) and macrophages (IL-6, TNFα) at 6 h post-infection compared to *BM* (*p* < 0.05). Interestingly, *BM*-induced the anti-inflammatory cytokine, IL-10, in ATII cells and macrophages at 6 h post-infection, with delayed induction of inflammatory cytokines at 24 h post-infection. Because *BP* is flagellated and produces LPS, we confirmed that it stimulated both Toll-like receptor (TLR) 4 and TLR5 via NF-κb activation while the non-flagellated *BM* stimulated only TLR4. These data show the differences in *BP* and *BM* pathogenicity in the lung when infecting human ATII cells and macrophages and demonstrate the ability of these pathogens to elicit distinct immune responses from resident lung cells which may open new targets for therapeutic intervention to fight against these pathogens.

## Introduction

*Burkholderia pseudomallei* (*BP*) and *Burkholderia mallei* (*BM*) are facultative-intracellular Gram-negative bacilli that cause melioidosis and glanders, respectively (White, 2003; Gilad, 2007; Galyov et al., 2010). *BP* is a highly motile pathogen (Dance, 1991; Leelarasamee, 2004; Lazar Adler et al., 2009; Wikraiphat et al., 2009) that causes melioidosis in animals and humans (White, 2003; Gilad, 2007). The clinical symptoms of *BP* are difficult to diagnose because they present as flu-like symptoms, pneumonia, or septicemia (Dance, 1991; Leelarasamee, 2004; De Keulenaer and Cheng, 2006; Cheng et al., 2007). Melioidosis is endemic in subtropical and tropical areas, with most cases found in Southeast Asia and northern Australia where it is the most common cause of community-acquired pneumonia (White, 2003; Gilad, 2007; Galyov et al., 2010). A pulmonary *BP* infection can disseminate to distal organs with consequential development of bacteremia that can lead to death (White, 2003; Lazar Adler et al., 2009). In contrast, the closely related *BM* is a non- motile pathogen that is the etiological agent of glanders that persists in its equine host to survive (Redfearn et al., 1966; Gilad, 2007; Galyov et al., 2010). If transmitted to humans, the clinical signs of the disease are febrile pneumonia accompanied resulting in dissemination from the lungs to distal organs causing bacteremia and necrosis (Redfearn et al., 1966; Gilad, 2007; Galyov et al., 2010). Therefore, both of these pathogens are considered viable candidates for use as bioweapon agents via aerosolization effecting a large populated area.

*BP* and *BM* are phylogenetically very similar containing nearly identical 16S ribosomal DNA sequences with genome variations between the strains (Godoy et al., 2003; Holden et al., 2004). The *BP* genome contains two chromosomes, a large chromosome of 4.07 Mb that carries genes associated for metabolism and growth, and a smaller chromosome (3.17 Mb) encoding genes responsible for environmental adaptation and bacterial survival including the type III secretion system, capsule, and flagellum (Holden et al., 2004). *BM* also has two chromosomes but has a downsized genome. Chromosome 1 (3.5 Mb) encodes for genes involved in exopolysaccharide capsule, LPS, type IV pili, and type III and IV secretion systems (Holden et al., 2004) while chromosome 2 (2.3 Mb) carries genes encoding for LPS biosynthesis, capsule production and bacterial metabolism (Holden et al., 2004). Unlike *BP*, *BM* is non-flagellated and non-motile due to IS elements that disrupts the fliP gene, an essential gene for flagellum biogenesis, and a frameshift mutation in the flagellum motor gene that eliminates its function (Holden et al., 2004).

Although both pathogens share some antigenicity and virulence-associated similarities their disease outcomes are quite unique. While both *BP* and *BM* have been shown to readily adhere and invade transformed human and murine macrophage-like cell lines (Jones et al., 1996; Stevens and Galyov, 2004; Pudla et al., 2011) and ATII cell lines (Kespichayawattana et al., 2004; Utaisincharoen et al., 2004, 2005; Boddey et al., 2006; Sim et al., 2009; Whitlock et al., 2009; Balder et al., 2010), relatively few studies have examined the immunological outcomes-induced following infection of primary human cells (Nathan and Puthucheary, 2005; Puthucheary and Nathan, 2006) or performed direct comparisons of their differential infectivity process. This is likely due to the level 3 bio-containment facilities necessary to perform experiments with *BM* and *BP*. With the atmosphere of uncertainty following the tragic events of September 11th, 2001 the potential for these highly pathogenic organisms to be used as a biological weapon via aerosolization is a real possibility. Therefore, to gain a better understanding of *BM* and *BP* pathogenesis in the lung, we examined the induction of innate immune responses by primary human pulmonary cells in the early stages of *BM* or *BP* infection. The lung is constantly exposed to harmful inhaled environmental factors (i.e., bacteria, viruses, and chemicals). Pulmonary defenses have evolved such that the physiological positioning of resident alveolar cells are able to clear and/or fight against harmful particles. For example, alveolar macrophages (AM) are considered the first line of defense against inhaled pathogens. Successful pathogenic bacteria have developed mechanisms to subvert host defenses. Murine studies with *BP* showed macrophage invasion without activation of inducible nitric oxide synthase responsible for production of nitric oxide crucial to host attacks on intracellular bacteria (Utaisincharoen et al., 2001). The studies reported by Puthucheary and colleagues (Nathan and Puthucheary, 2005; Puthucheary and Nathan, 2006) indicated that *BP* was able to survive and replicate more successfully in macrophages collected from patients with melioidosis than normal macrophages from healthy volunteers. Additionally, Brett et al. (Balder et al., 2010) reported that *BM* LPS was a potent stimulator of cytokine secretion from human monocyte-derived macrophages (MDM) via TLR4, however, these studies did not examine the intact live pathogen. The interaction of *BM* with primary human macrophages is less studied and poorly understood.

Another residential pulmonary cell that will inevitably come into contact with inhaled *Burkholderia* is ATII cells, that represent approximately 4–5% of the cells in the alveolar space but comprises 15% of all the cells in the lung (Crapo et al., 1982, 2000; Novick et al., 1996; Mason, 2006). These non-professional immune cells are absolutely necessary for maintaining pulmonary homeostasis, specifically via the secretion of surfactants (Novick et al., 1996; Wright, 2005; Mason, 2006; Halliday, 2008; Pudla et al., 2011). ATII cells also are crucial, unconventional contributors to innate immunity via cytokine secretion, specifically chemokines and signaling molecules for professional immune cells including AM (O'Brien et al., 1999; Pechkovsky et al., 2000, 2005; Gentry et al., 2007). We have shown that the lethal pathogen, *Francisella tularensis*, stimulated polarized, primary human ATII cells to secrete chemotactic mediators that induced immune migration (Gentry et al., 2007). While there are various publications that describe *BM* (Whitlock et al., 2009) and *BP* (Stevens and Galyov, 2004; Utaisincharoen et al., 2005; West et al., 2008) interactions with non-polarized, transformed human A549 ATII cells, there are no reports using human primary ATII cells. Thus to remain physiologically, we utilized our *ex vivo* primary human ATII cell model as well as human MDM in the current studies to examine the pathogenic differences between *BM* and *BP* as revealed through their interactions with these residential pulmonary cells to induce different innate immune responses. These studies revealed that in the early stages of infection, *BP*, but not *BM*, readily adhered to ATII cells and macrophages and stimulated both TLR4 and TLR5 via its LPS and flagellin respectively. *BP* also induced high levels of cytokine secretion from ATII cells and MDM in the first 6 h after infection. Conversely, upon initial infection, *BM*-induced an inflammatory suppressor, IL-10, from ATII cells and macrophages with delayed secretion of inflammatory cytokines via TLR4 at 20 h post-infection.

## Materials and methods

### Bacterial strains

*Burkholderia mallei* (*BM*) China 7 (3873, MM-B, NBL7) and *Burkholderia pseudomallei* (*BP*) China 3 (MP-H, NBL 104) were obtained from BEI Resources (Manassas, VA) and propagated overnight at 37°C in Luria-Bertani (LB) broth (Difco Laboratories, Detroit, MI) from frozen stocks. Bacterial titers were determined using a Bel-Art—KLETT™ Colorimeter (Bel-Art Products, Pequannock, NJ) and confirmed by plating serial dilutions on LB agar plates. Both *BM* and *BP* are CDC classified category A select agent thus they were used in UTMB's certified BSL3 facility as per CDC and Federal regulations.

### Human cell cultures

Primary human type II alveolar epithelial (ATII) cells (ScienCell, San Diego, CA) were seeded on BioCoat Growth Factor Reduced Matrigel®-coated transwell filters (BD; Franklin Lakes, NJ) and maintained using Bronchial Epithelial Medium enhanced with the Bullet kit supplements provided by the manufacturer (BEGM; Lonza, Basel, Switzerland) supplemented with 10% charcoal-stripped FBS (Hyclone; Logan, UT) and 10 ng/mL of keratinocyte growth factor (KGF; PeproTech, Rockhill, UT). ATII cells were grown to confluence (4–5 days) then used for experiments. Before each experiment, the A549 or ATII cells were washed 2X with appropriate medium containing no serum or antibiotics. The serum-free medium used during experimentation ensured no interference by serum proteins and did not adversely affect the health or metabolic activity of the cells.

Human monocytes were isolated from peripheral blood mononuclear cells obtained from healthy donors with full approval of the UTMB IRB. Monocytes were isolated using a Hypaque-Ficoll (GE Healthcare, Piscataway, NJ) density gradient (800 × g at 21°C) followed by selection using a human monocyte enrichment kit without cd16 depletion (Stemcell #19058) and Easysep magnet (Stemcell #18000) counterflow centrifugal elutriation (Beckman J2-21 M/E centrifuge with JE-B6 elutriator rotor, Beckman Instruments, Palo Alto, CA). The purified monocytes were cultured in RPMI 1640, 10% FBS supplemented with GM-CSF (100 ng/mL) for 7 days to ensure differentiation into macrophages as previously described (Eaves-Pyles et al., 2011). The macrophages were detached from the culture plate using a non-enzymatic cell dissociation solution (Sigma-Aldrich). MDM differentiation was confirmed by flow cytometry of cell surface markers expression (CD11b, CD80).

### Infection model and cytokine analyses

ATII cells or MDM (5 × 10^5^/condition) were infected with *BM* or *BP* at an MOI of 100:1. Un-stimulated cells were used as negative controls. At 6 h post-infection, supernatants were collected from ATII cells and MDM then frozen at −80°C until analysis. At the same time point, parallel ATII cells or MDM cultures were washed with 2 mL of PBS then incubated for 30 min with serum-free medium containing 50 ug/mL of gentamicin (Sigma). ATII cells and MDM were washed then resuspended in serum-free medium containing 5 ug/mL of gentamicin and incubated an additional 14 h at 37°C/5% CO_2_. Supernatants were collected at 20 h post-infection then 6 and 20 h supernatants were analyzed via individual human ELISA kits (Pierce-Endogen) for the following cytokines: IL-6, IL-8, IL-10, and/or TNF-α.

### Scanning electron microscopy (SEM)

ATII cells grown on transwell filters were exposed apically to *BM* or *BP* at an MOI of 100:1 for 4 h. Then filters were fixed in a mixture of 2.5% formaldehyde, 0.1% glutaraldehyde in 0.05 M cacodylate buffer pH 7.2 containing 0.03% trinitrophenol and 0.03% CaCl_2_, washed in 0.1 M cacodylate buffer pH 7.2 and post-fixed in 1% OsO_4_ in the same buffer. The filters were then dehydrated in ethanol and incubated with hexamethyldisalazane. After the filters were air-dried they were sputter coated for 40 s with iridium in an Emitech K575X turbo sputter coater (Emitech, Houston, TX). Samples were visualized using a Hitachi S4700 field emission scanning electron microscope (Hitachi High Technologies America, Electron Microscope Division, Pleasanton, CA) at 2 kV (UTMB Electron Microscopy Laboratory Facility).

### Bacterial adhesion and invasion assays

The ability of *BM* and *BP* to adhere to ATII cells was performed as previously described (Eaves-Pyles et al., 2008). Briefly, the cells were grown on 24-well plates to confluence at 37°C in 5% CO_2_ then infected with BM or BP at an MOI of 100:1 for 4 h at 37°C. To quantify adherence of the pathogens, the infected cells were washed twice with PBS then lysed with 200 μl of 0.1% Triton X-100 in PBS buffer. The adherent bacteria were plated, incubated and then colonies were counted the following day. To obtain accurate counts of the pathogen that adhered to ATII cells, bacterial invasion experiments were performed in parallel to the bacterial adhesion. Then the number of bacteria that invaded the cells was subtracted from the number of bacteria that adhered to the cells. Therefore, pathogen invasion was determined by infecting ATII cells with *BM* or *BP* as described above. Four hours post-infection, cells were washed twice with sterile PBS and then incubated with DMEM containing 50 ug/mL gentamicin for 1 h to kill extracellular bacteria. Cells were washed with PBS, lysed with 0.1% Triton X-100, plated and colonies were counted the following day as above.

### Macrophage phagocytosis and intracellular replication of *BM* and *BP*

MDM (5 × 10^5^ cells) were stimulated with *BP* or *BM* (MOI 100:1). To examine phagocytosis and intracellular replication of *BM* and *BP*, at 1 h post-infection, macrophages were washed with 2 mL of PBS then incubated for 30 min with serum-free medium containing 50 ug/mL of gentamicin (Sigma) to kill extracellular bacteria (Eaves-Pyles et al., 2001a). Cells were lysed by adding 0.1% SDS and lysates were plated on BHI plates and incubated at 37°C in 5% CO_2_ overnight to allow quantification of bacterial phagocytosis. To quantify intracellular replication of the pathogens, parallel macrophage cultures were treated and infected as described above. However, following the 30 min gentamicin incubation, cells were washed then resuspended in serum-free medium containing 5 ug/mL of gentamicin. Cells were incubated an additional 3 h at 37°C/5% CO_2_ then lysed, plated, and single colonies were counted as described above.

### TLR stimulation by *BM* or *BP*

As described previously (Gentry et al., 2007), human embryonic kidney (HEK) 293 cells genetically manipulated to express human TLR4, or TLR5 (Invivogen, San Diego, CA) were cultured and maintained in DMEM (Cellgro Mediatech) supplemented with 10% FBS, 100 units/mL pencillin, 100 ug/mL streptomycin, 10 ug/mL basticidin, and 2 mM glutamine. To analyze activation of the transcription factor NF-κb, each of HEK293 cell lines were transfected with the pNF-κB-secreted alkaline phosphatase (pNF-κB-SEAP) reporter plasmid (Clontech, Mountain View, CA) using Lipofectamine Plus 188 (Invitrogen) per the manufacturer's instructions. The day after transfection, the existing medium was replaced with fresh DMEM containing no antibiotics followed by the addition of *BM* or *BP* at an MOI of 100:1. Commercial TLR agonists (Invivogen) were used as positive controls that included LPS (TLR4) and flagellin (TLR5). Medium alone and HEK293 cells with no agonist served as negative controls. The cells were incubated for 6 h then medium was aspirated and replaced with fresh DMEM with antibiotics and incubated overnight at 37°C and 5% CO_2_. Collected Supernatants were heat inactivated for 30 min at 65°C. SEAP activity in each sample was quantified (Mason, 2006) by the Phospha-Light Chemiluminescence Reporter Assay (Tropix, Bedford, MA) and a TR717 microplate 195 luminometer with WinGlow software (Tropix/PE Applied Biosystems, Bedford, MA196).

### Western immunoblot

Western blots were performed as we described previously (Eaves-Pyles et al., 2001a,b). Briefly, supernatants from *BM* and *BP* (as collected above) were analyzed for the amount of protein in each sample by the Bradford assay (Bio-Rad, Hercules, CA). The supernatants were boiled in loading buffer [4% SDS, 20% glycerol, 125 mM Tris-HCl (pH 6.8), and 10% 2-mercaptoethanol] then 40 μg of protein was loaded on an 8–16% Tris-glycine gradient gel (Novex, San Diego, CA). Electrophoresed proteins were transferred to a nitrocellulose membrane (Novex) and membranes were blocked with 10% nonfat dried milk for 30 min prior to incubation with rabbit polyclonal anti-(FliC) flagellin (BioLegend, San Diego, CA) at a dilution of 1:1000 overnight. Blots were washed, followed by the addition of peroxidase-conjugated anti-rabbit immunoglobulin G (Sigma Chemical Co., St. Louis, MO) at a dilution of 1:10,000 for 3 h. Blots were washed and then incubated for 1 min in enhanced chemiluminescence reagents (ECL kit; Amersham, Little Chalfont, Buckinghamshire, England). Processed blots were placed on X-ray film (Kodak®) for empirically optimized exposures.

### Statistical analysis

Numerical results are presented herein as mean ± SEM of two or three-independent experiments containing replicate experimental conditions per each experiment. Statistically analysis of numerical data was completed by Student's *t*-test or analysis of variance (ANOVA) using Prism software (Graph Pad v4.0, San Diego, CA). Differences were noted to be statistically significant when the *p* value was < 0.05.

## Results

### *BP* and *BM* interact with the apical surface of ATII

**Figure 1:**
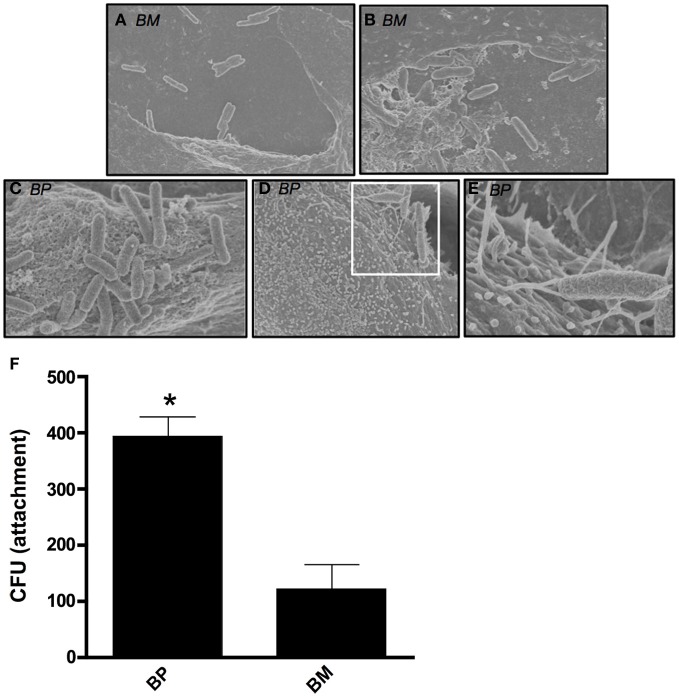
***B. mallei* (*BM*) and *B. pseudomallei* (*BP*) interaction with primary human ATII cells (ATII cells).** ATII cells were grown on 3.0-um transwell filters to confluence prior to apical exposure of *BM* and *BP* at an MOI of approximately 100:1 for 4 h. Minimal contact of *BM* (**A** and **B**) with ATII cells was visualized via SEM. However, *BP* **(C–E)** adhesion to the apical surface of ATII cells was observed and the pathogen was entangled by the ATII microvilli [**D** (white boxed area) and **E**]. Further, quantitative analysis of attachment showed that BP significantly adhered better to ATII cells compared to BM (*p* < 0.05; **F**). Data is representative of triplicate samples of three experiments and is represented as mean ± SEM. ^*^p < 0.05.

We sought to visualize the interaction between *BM* or *BP* and ATII cells during early stages of a pulmonary infection. To remain biologically and physiologically relevant, ATII cells were grown on transwell filters to allow polarization before being infected apically with *BM* or *BP*. Four hours post-infection, scanning electron microscopy (SEM) was used to visualize interactions between the bacteria and the ATII cells. Figure 1 illustrates the distinct differences between *BM* and *BP*'*s* contact with the ATII cells. *BM* contacted the apical surface of ATII cells but did not appear to readily adhere to the ATII cells at 4 h post-infection as such the pathogen could be seen floating free in the medium (Figures 1A,B). Conversely, *BP* consistently established intimate contact with the apical surface of ATII cells (Figures 1C–E) where ATII microvilli are wrapped around individual *BP* organisms (Figures 1D,E). In addition to these findings, quantitative analysis of pathogen adhesion to ATII cells showed that at 4 h post-infection *BP* adhered to the apical surface of ATII cells significantly better than BM (*p* < 0.05; Figure 1F). These data demonstrate distinct differences between *BM* and *BP* as the efficient adherence of *BP* to ATII cells is more pronounced in the early stages of infection than *BM*'s ability to bind to host cells.

### *BM* and *BP*-induced distinct as well as time-dependent cytokine secretion from ATII cells and MDM

**Figure 2:**
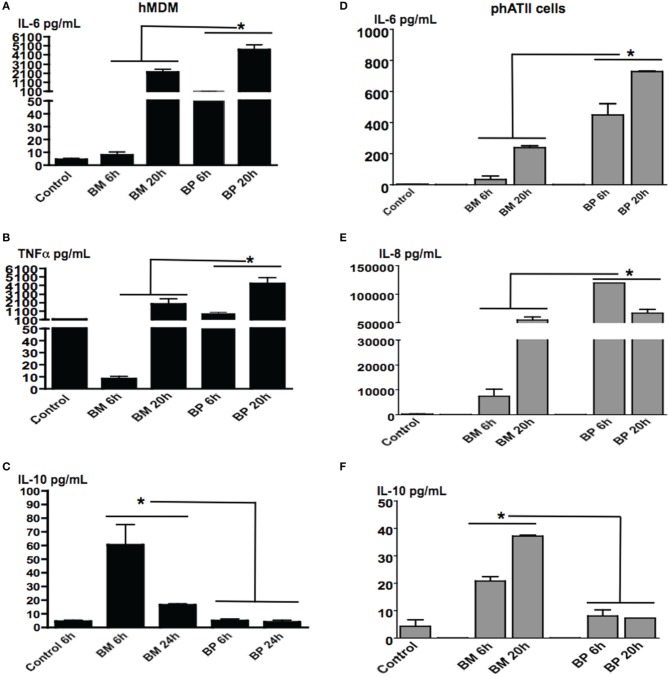
***BM* and *BP*-induced time-dependent secretion of cytokines from ATII cells and MDM.** ATII cells and MDM were infected with *BM* or *BP* (MOI 100:1) for 6 or 20 h. Supernatant was collected and analyzed for cytokine secretion. Un-stimulated cells served as baseline controls. *BP* stimulated higher levels of IL-6 **(A)** and TNF-α **(B)** from MDM as well as IL-6 **(D)** and IL-8 **(E)** from ATII cells at 6 and 20 h post-infection compared to *BM*. However, *BM*-induced significantly higher IL-10 secretion from MDM compared to *BP* at 6 h post-infection **(C)**. Additionally, IL-10 was significantly secreted from BM-ATII cells compared to BP at both 6 and 20 h post-infection **(F)**. Data is representative of two experiments containing duplicate conditions/experiment and is represented as mean ± SEM. ^*^denotes significance of *p* < 0.05.

Both ATII cells and AM will be contacted by inhaled *BM* and *BP* over the course of pulmonary infections. As such, we sought to evaluate the responses of these residential professional immune cells and epithelial cells responded to the pathogens in different the stages of infection. MDM and ATII cells were infected with *BM* or *BP* at an MOI of 100:1 then cells supernatants were collected at 6 and 20 h post-infection. Results showed that *BP* stimulated significantly higher levels of cytokine secretion from MDM [Figures 2A (IL-6), B (TNFα)] and ATII cells [Figures 2D (IL-6) and E (IL-8)] at both tested time points compared to *BM* or uninfected controls (*p* < 0.05). At the later time point it was observed that *BM* provoked responses. Specifically, secretion of IL-6 and TNF-α from MDM and IL-6 and IL-8 from ATII cells increased at 20 h post-infection (Figures 2A,B,D,E). Interestingly, the anti-inflammatory cytokine, IL-10 was secreted by ATII cells and MDM to significantly higher levels at 6 h post-*BM* infection compared to *BP*, then IL-10 levels decreased by 20 h post-infection, but remained higher than uninfected controls and *BP*-infected cells (*p* < 0.05; Figures 2C,F).

These data demonstrate that *BP* and *BM* induce distinct cytokines that benefits their specific survival in ATII cells and macrophages. In the initial stages of infection, *BP*-induced vigorous immune responses in both cell types, while *BM*-induced an anti-inflammatory response (i.e., IL-10) in the early stages of infection then transitioned into a traditional immune response.

### Phagocytosis and intracellular replication of *BM* and *BP*

**Figure 3:**
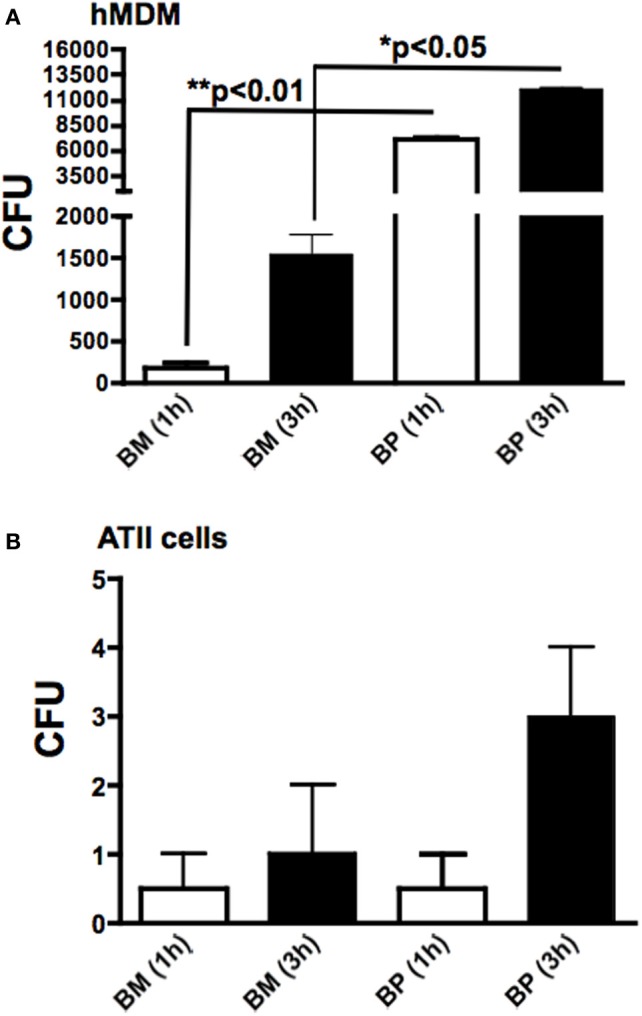
**Phagocytosis and intracellular replication of *BM* and *BP*.** MDM and ATII cells were infected with *BM* or *BP* (MOI of 100:1). As determined by CFUs, phagocytosis (accessed at 1 h) and intracellular replication (accessed at 3 h post-infection) of *BP* by MDM was significantly higher compared to BM **(A)**. Alternatively, neither *BM* nor *BP* invaded or replicated to high numbers in ATII cells **(B)**. Data is representative of two experiments containing duplicate conditions/experiment and is represented as mean ± SEM. ^*^denotes significance of *p* < 0.05 and ^**^p < 0.01.

Because *BM* and *BP* interacted with MDM and ATII cells to induce cytokines, we sought to determine the invasion capabilities and intracellular replication of *BM* and *BP* in these host cells. To this end, MDM and ATII cells were infected with *BM* or *BP* (MOI 100:1) for 1 h to determine phagocytosis of the pathogens and at 3 h post-infection to assess intracellular replication. Cells were lysed at each time point, plated then the bacterial CFU were quantified. Several observations were made from these data results. First, *BP* was more readily phagocytosed at 1 h post-infection by hMDM (Figure 3A) compared to phATII cells (*p* < 0.05; Figure 3B). However, *BP* replicated in both cell types to relatively high numbers at 3 h post-infection (Figures 3A,B). Second, the phagocytosis of *BM* by hMDM and ATII cells was minimal (Figures 3A,B) but the intracellular replication of *BM* in hMDM, but not in phATII cells, was considerably high at 3 h post-infection (Figure 3A).

The culmination of these data showed that the professional immune cell, macrophages, markedly surpassed the resident pulmonary epithelial cells, ATII cells, in phagocytosing *BP* and to a lesser extent *BM.* Both pathogens replicated rapidly to high numbers in macrophages suggesting bacterial manipulation and/or evasion of innate defenses.

### Stimulation of TLRs by *BM* and *BP*

**Figure 4:**
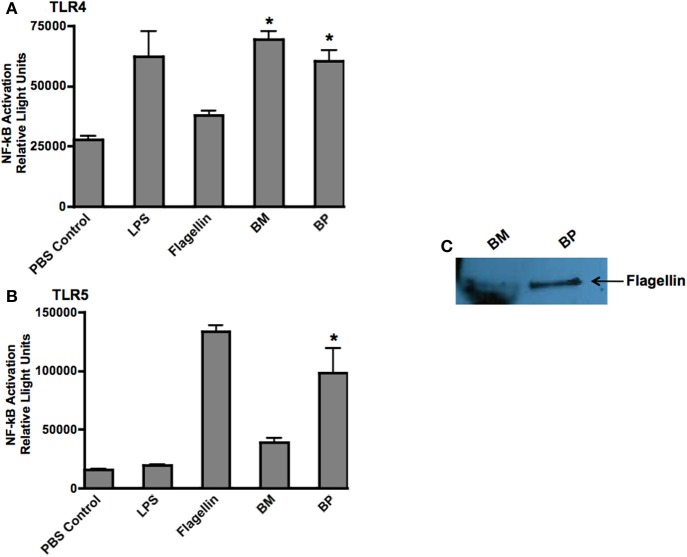
***BM* and *BP* stimulation of various Toll-Like Receptors.** To determine if *BM* and/or *BP* stimulated various TLRs, HEK293 cells expressing TLRs 4 and 5 and transfected with the firefly luciferase plasmid pNiFty2-Luc were stimulated with *BM* or *BP* at an MOI of 100:1. Un-stimulated cells served as negative controls. The TLR agonist served as positive controls. The cells were incubated with live bacteria for 6 h. The medium was then aspirated and replaced with fresh DMEM without antibiotics and incubated overnight at 37°C and 5% CO_2_. The TLR agonists remained with the cells for an overnight incubation. The following day, samples were analyzed to determine luciferase activity. Data is expressed as relative luciferase activity. Both *BM* and *BP* **(B)** stimulated TLR4 as effectively as the LPS agonist **(A)**. However, the flagellin from *BP* stimulated TLR5 **(B)** as we detected flagellin in the medium of B-infected cells but not *BM* **(C)**. Three experiments were performed in using triplicate samples and data is represented as mean ± SEM and ^*^denotes significant differences of *p* < 0.01.

Because TLRs are at the forefront of recognizing invading pathogens resulting in the up-regulation of innate immune response via the NF-κb signal transduction pathway (Hatada et al., 2000; O'Neill and Bowie, 2007), we investigated the ability of *BP* and *BM* to stimulate TLR4 and TLR5. We chose TLR4 because it is the receptor for LPS and both pathogens are Gram-negative microbes. Additionally, because *BP*, but not *BM*, is flagellated, we hypothesized that *BP* flagellin would stimulate TLR5. Stability transfected HEK293 cells expressing human TLR4 or TLR5 were co-transfected with the NF-κb-SEAP reporter plasmid then infected with *BP* or *BM* at an MOI of 100:1. Following an overnight incubation, supernatants were collected and analyzed for SEAP secretion. Un-stimulated cells were used as negative controls while additional cultures of HEK293 TLR4 and TLR5 expressing cells were stimulated with their respective TLR agonist (purified LPS for TLR4 or flagellin for TLR5), which served as positive controls. Our results showed that both *BP* and *BM*-induced NF-κb activation via TLR4 stimulation that was equivalent to purified LPS and significantly higher than flagellin or un-stimulated controls (*p* < 0.05; Figure 4A). However, as predicted, only the flagellated *BP*-induced NF-κb via TLR5 stimulation compared to *BM* and LPS (*p* < 0.05; Figure 4B). To confirm the release of *BP* flagellin into the surrounding environment, immunoblot analysis detected the presence of flagellin in the *BP* supernatant compared to no detection of flagellin in the supernatant of *BM* (Figure 4C).

These findings demonstrate that *BP*, but not *BM*, possesses various virulence factors that readily stimulate multiple TLRs thereby rapidly inducing innate immune responses.

## Discussion

The importance of studying the pathogenesis of inhaled *BM* and *BP* has become more imminent as their potential for agents of bioterrorism has become known. Although *BM* and *BP* are in the same genus of bacteria, in this current study our findings showed that they demonstrate distinct pathogenicity characteristics upon the infection of resident lung cells, providing better understanding of their different disease outcomes.

Pathogen adhesion to host epithelial cells offers an opportunity for invasion and ultimately colonization of the host. It has been shown that both *BM* and *BP* possess certain genes that aid in host cell adherences such as *boaA* adhesion genes while others are exclusive to *BP* such as type IV pili, type III secretion system, flagella (Inglis et al., 2003; Kespichayawattana et al., 2004; Boddey et al., 2006; Balder et al., 2010). Flagella are recognized as important indirect contributors to bacterial virulence as the motility provided by the flagellum structure is frequently associated with the ability of a pathogen to move through its surrounding environment, as well as aid in attachment, adhesion, and invasion to host cells subsequently contributing to disease processes (Komoriya et al., 1999; Eaves-Pyles et al., 2001a; Chua et al., 2003; Ramos et al., 2004; Chuaygud et al., 2008). Our findings showed that the flagellated *BP*, but not the non-flagellated *BM*, readily adhered to ATII cells in the first 4 h post-infection. *BP*'*s* motile and adhesion characteristics provided by flagella is likely allowing a more intimate interaction with host cells, facilitating the phagocytosis of the bacteria, while non-flagellated *BM* had minimal cellular adhesion in the early stages of infection. *BP* flagellum was shown to be necessary to penetrate the mucous lining the respiratory tract in order to gain access to host cells in intranasal and intraperitoneal infection mice models (Chua et al., 2003). Other studies have shown that an aflagellated *BP* mutant was internalized significantly less by macrophages and ATII cells as compared to wild type *BP* (Chuaygud et al., 2008). Likewise, flagellin negative variants have been observed to be less endocytosed than wild type flagellated *BP*, which signifies the involvement of the *BP* flagellum in cellular invasion (Inglis et al., 2003).

While the intact flagella structure contributes to the adherence and invasion of a pathogen to host cells, it is the primary protein component of the flagellum structure, known as flagellin, that is now recognized as a major virulence factor of Gram-negative organisms (Eaves-Pyles et al., 2001a,b, 2011; Chua et al., 2003; Ramos et al., 2004; Chuaygud et al., 2008). The release of flagellin monomers into the surrounding environment occurs when the flagella are shed from the bacteria and the structure disassembles due to environmental and chemical factors or deliberate ejection from the bacteria (Ramos et al., 2004). Only after flagellin monomers are free from the flagella filament do they bind to and stimulate signaling pathways via their receptor, TLR5 (Smith et al., 2003), resulting in the secretion of inflammatory cytokines (Eaves-Pyles et al., 2001a,b, 2011). As such, we detected flagellin in the supernatant of *BP*, but not *BM*, of infected cultures, leading to NF-κb activation via TLR5 stimulation. Additionally, *BM*, as well as *BP*, stimulated TLR4 as both pathogens possess LPS as a major outer membrane component. Our findings correlate with other studies demonstrating the ability of *BP* to stimulate TLR4 and TLR5 (Hii et al., 2008; West et al., 2008; Wiersinga et al., 2008) and *BM*'*s* stimulation of TLR4 only (Brett et al., 2007). Thus it is reasonable to conclude that as multiple TLRs are subject to stimulation by *BP*, but not *BM*, this likely contributed to the vigorous innate immune responses (e.g., cytokine secretion) induced by macrophages and ATII cells in the early stages of infection. Sim et al. (2009) showed that primary murine epithelial lung cells both *in vitro* and *in vivo* secrete significant levels of pro-inflammatory cytokines in response to *BP*. Our findings, as well as others (Stevens and Galyov, 2004; Sim et al., 2009), resemble the clinical situation of acute melioidosis in that this disease can lead to induction of pro-inflammatory cytokines with high levels of cytokine secretion, leading to poorer prognosis for infected patients (Simpson et al., 2000; Cheng et al., 2007; Wiersinga et al., 2007). Alternatively, and very interestingly, *BM* stimulated minimal cytokine secretion from macrophages and ATII cells in the initial infection stage but stimulated the secretion of pro-inflammatory suppressor, IL-10. However, cytokine secretion from ATII cells and macrophages increased by 20 h post-*BM* infection. A study by Brett et al. (2008) found that *BM* was a weak inducer of cytokines, including IL-10, from a macrophage cell line. Although both studies demonstrate a lack of cytokine secretion by *BM*-infected macrophages, the differences (i.e., IL-10 secretion) between these findings may be explained by the fact that our studies used primary human monocytes-derived macrophages in suspension while Brett et al. (Cheng et al., 2007) tested monolayers of a murine macrophage-like cell line infected with *BM*. As a result of our findings, we may hypothesize that the lack of inflammatory cytokine secretion but increased secretion of IL-10 by macrophages and ATII cells is a mechanism evolved by *BM* to avoid detection by the host. This evasion by *BM* would allow the pathogen time to adapt to the intracellular environment and replicate before the host detects it and is able to mount an effective, timely immune response.

Because alveolar macrophages are at the forefront of the host's immune defenses, it was not surprising that macrophages secreted higher levels of cytokines overall in the response to *BM* and *BP* compared to ATII cells. Additionally, although *BP* readily adhered to ATII cells, there was minimal invasion of and replication in ATII cells by both pathogens. Other studies have shown that *BP* adhere, invade, and replicate to adequate numbers in transformed ATII cell lines (Whitlock et al., 2009; Balder et al., 2010). However, our studies herein are the first to examine *Burkholderia* pathogenesis using primary human alveolar type II cells in a physiological relevant *ex vivo* model. While ATII cells are not professional immune cells, they contribute significantly to the pulmonary immune defenses against inhaled invaders via cytokine secretion and surfactant A and D (Crapo et al., 2000; Wright, 2005; Lhert et al., 2007; Chroneos et al., 2010). Our results suggest that ATII cells are an important cytokine secreting cells resulting from interactions with *BM and BP*, however, they are not a reservoir of invasion and intracellular replication for these pathogens, which may open new avenues of the host innate immune response for therapeutic treatment of melioidosis and glanders.

In conclusion, we have shown various distinct differences between *BM* and *BP* pathogenesis regarding their interaction with primary human ATII cells and macrophages. *BM* and *BP* are similar in various aspects of their pathogenesis they produce different disease outcomes. Our study demonstrates that each of these pathogens has several unique strain specific characteristics is revealed in their interactions with host cells to induce varied host immune responses. By delineating host immune responses to *BM* and *BP* we can better understand the pathogenesis of these pathogens and develop new directions for therapeutic intervention and vaccines.

### Conflict of interest statement

The authors declare that the research was conducted in the absence of any commercial or financial relationships that could be construed as a potential conflict of interest.
